# An Efficient Weighted Graph Strategy to Identify Differentiation Associated Genes in Embryonic Stem Cells

**DOI:** 10.1371/journal.pone.0062716

**Published:** 2013-04-26

**Authors:** Jie Zhang, Li Li, Luying Peng, Yingxian Sun, Jue Li

**Affiliations:** 1 Department of Prevention, Tongji University School of Medicine, Shanghai, China; 2 Key Laboratory of Arrhythmias, Ministry of Education, Tongji University School of Medicine, Shanghai, China; 3 Department of Cardiology, The First Hospital of China Medical University, Shenyang, China; Universitat Politecnica de Catalunya, Spain

## Abstract

In the past few decades, embryonic stem cells (ESCs) were of great interest as a model system for studying early developmental processes and because of their potential therapeutic applications in regenerative medicine. However, the underlying mechanisms of ESC differentiation remain unclear, which limits our exploration of the therapeutic potential of stem cells. Fortunately, the increasing quantity and diversity of biological datasets can provide us with opportunities to explore the biological secrets. However, taking advantage of diverse biological information to facilitate the advancement of ESC research still remains a challenge. Here, we propose a scalable, efficient and flexible function prediction framework that integrates diverse biological information using a simple weighted strategy, for uncovering the genetic determinants of mouse ESC differentiation. The advantage of this approach is that it can make predictions based on dynamic information fusion, owing to the simple weighted strategy. With this approach, we identified 30 genes that had been reported to be associated with differentiation of stem cells, which we regard to be associated with differentiation or pluripotency in embryonic stem cells. We also predicted 70 genes as candidates for contributing to differentiation, which requires further confirmation. As a whole, our results showed that this strategy could be applied as a useful tool for ESC research.

## Introduction

Embryonic stem cells (ESCs) are unspecialized cells that have the ability of self-renewal, producing daughter cells with equivalent developmental potential, or to differentiate into more specialized cells. Experiments performed several decades ago showed that dormant gene expression programs can be awakened in differentiated cells by the fusion of different pairs of cell types [1]. Different cell fates can be induced by the defined transcription factors [2]. However, the global transcription activities in ESCs are not well understood, and the set of differentiation associated genes, i.e. the genes which are active in the pluripotent state and become inactive upon differentiation (and vice versa), is still unknown.

Rapid increase of high throughput biological data supplies us both opportunities and challenges to explore mechanisms in ESCs differentiation. In fact, initial approaches derive predictions based on specific information such as gene expression profile [3] and protein-protein interactions [2]. Also, it has been shown that the use of global optimization may not actually yield significant improvement over simpler local prediction methods [4], [5], [6].Here, we propose an intuitive method, which uses a unified framework for combining multiple sources, including mRNA expression profile dataset, sequence dataset and protein-protein interaction dataset. Our method involves three steps. Firstly, each evidence source is assessed with a reliability score based on their functional correlation. According to the data characteristics, a weighted value is defined. Secondly, undirected graphs are constructed based on each data source respectively, with genes as vertices and functional relationships between gene pairs as edges. Finally, these undirected graphs are integrated into a weighted functional linked network. The genes are predicted to be differentiation associated genes based on their degrees in the final network, which are regarded to be associated with differentiation or pluripotency in embryonic stem cells.

Our results showed that despite the simplicity of its formulation, our method performed relatively well on the prediction ability of identifying the differentiation associated genes. It was also shown that our method could involve a large amount of datasets, including cross genome information, in order to make much better predictions.

## Materials and Methods

### Datasets Preprocessing and Normalization

Four different types of datasets were analyzed. The Affymetrix mouse stem cell microarray data (GSE7506) consisted of 36 samples, which were used for prediction and testing of novel networks regulating ESCs self-renewal and commitment [7]. It was pre-processed by Robust Multi-array Analysis (RMA) followed by median normalization between arrays [8]. The protein sequences were downloaded from RefSeq database containing a total of 38129 distinct sequences (June 11, 2010). Functional annotations were taken from Gene Ontology (GO) (June 20, 2010). The annotations were arranged in a hierarchical manner and compiled using up-to-date information from GO’s three ontology divisions, including Molecular Function (MF), Biological Process (BP) and Cellular Component (CC). The mouse protein-protein interaction (PPI) datasets (October 10, 2010) were downloaded from APID [9], BIND [10], iRefIndex [11], MINT [12] and STRING [13], which contained 12026, 8164, 19727, 4333 and 207211 PPIs respectively. To increase the coverage of the PPI network, the five datasets were pooled together as previously done in Lage et al. [14].

### Selection of Differentially Expressed Genes

We used the popular SAM (Significance Analysis of Microarrays, samr R package) method [15] to select differentially expressed genes (DEGs). Multiple statistical tests were controlled by false discovery rate (FDR) defined as the expected percentage of false positives among the claimed DEGs [16]. Because the FDR estimation of SAM might be overly conservative [17], [18], we also applied the FDR estimation method suggested by Zhang [18] using the idea of Xie [17], and refer to it as the modified SAM method.

### Scoring Functions

The weighted graph strategy utilized different weighted scores as inputs. According to the character of different dataset, we applied a simple weighted strategy similar to the weighted averages method [19].

1. Gene expression profiles. Relationship between gene *i* and *j* was scored based on Pearson Correlation Coefficient (PCC) by the expression profiles, denoted as 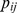. An average of 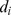 and 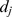 which are SAM statistics [18] is assigned as the weight of 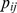. The weighted score was defined as formula 1. To scale the score between 0 and 1, the score of each gene pair was divided by 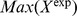 which was the highest value of all gene pairs.

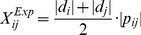
(1)

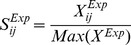
(2)
2. Sequence analysis. Each mouse sequence was aligned with all other sequences using software ClustalX [20]. The identity matrix was applied to calculate scores 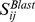 which represented the sequence similarities of each two amino acids. And the score was automatically adjusted to positive values, scaled between 0 and 1.
3. GO functional analysis. The pair-wise functional similarities of the DEGs were computed and analyzed. Each gene was represented by a feature vector containing the gene’s similarities to predefined prototype genes. The scores between gene *i* and *j* were calculated in molecular function, biological process and cellular component respectively. For scaling the score between 0 and 1, 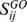, representing the semantic similarity between gene *i* and *j* [21], [22], [23], was calculated as formula 3.

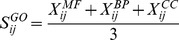
(3)

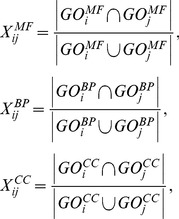
(4)

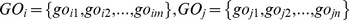
(5)

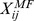
, 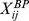 and 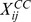 measured the functional similarities of three basic ontology divisions between gene *i* and *j* (Formula 4). 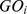 and 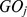 were two sets of GO terms that annotated with gene *i* and *j* respectively (Formula 5).
5. Protein-protein interactions. The FSWeight [24] has been shown to provide a good estimate of functional similarity between the interacting protein pairs (direct interactions), as well as between the protein pairs that do not interact, but share common interaction partners (indirect interactions). To keep our comparison simple, we only used direct interaction pairs. Each interacting protein pair was scored using a simplified variant of the FSWeight measurement (Formula 6), where 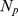 referred to the set that contains 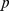 and its interaction neighbors. 
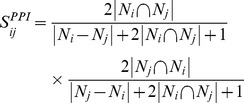
(6)

### Combination of Different Datasets

The initial four score matrixes of four datasets just included DEGs respectively. Lee et al. [25] used a unified log-likelihood scoring function to combine several sources of binary gene relationship data into a graph, which could be clustered into groups that show strong similarity in function. It has been illustrated that different data sources have different degrees of correlation with function similarity. Here, we adapted a simple model in our approach to integrate the four datasets. Each dataset can be modeled as an undirected graph, where each vertex represents a protein and each edge represents the functional relationship between proteins. The edges in different graphs have different scoring schemes as previously described. Different graphs derived from four score matrixes were combined to form a larger and presumably more complete graph. The confidence relationship of each edge in the last complete graph can be estimated by an integrated score, which represents a particular function shared between two genes. The score of the two proteins in the final integrated graph can be calculated as formula 7.
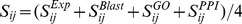
(7)


### Generation of Differentiation Associated Genes

The final network was built with the gene pairs if their scores were larger than the 75% quantile of the whole score values (Formula 7), since when the threshold was higher than 75%, some differentiation associated genes would not be selected and when it was lower than 75%, too many redundant genes would be selected. In a network, nodes with high connectivity were more important than low connectivity. They were named as “hubs”. A line graph showed the relationship between degree and gene number. According to the chart, genes with most of higher degrees were selected, which were considered as differentiation associated genes.

### Validation Method

For comparison, we ran three separate methods, SAM (using the mRNA expression data set) [18], decision tree (DT) [26] and normal graph strategy (NGS). In normal graph strategy, scores were calculated just based on Pearson Correlation Coefficients, blast scores, GO scores (same as 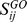) and the PPI scores (1 representing that the protein *i* interacts with protein *j*, 0 representing non-interacting proteins). The selected differentiation associated genes were predicted as positive gene set using three repetitions of 5-fold cross-validation. The area under Receiver Operating Characteristics [27] graph was computed for each class (associated or not associated with differentiation) and the average was obtained based on the predictions 15 times in total.

## Results

### Differentially Expressed Genes Selection

Current FDR control procedures, including the one adopted in SAM [15], may be unstable in small samples especially in the presence of correlated expression changes. Hence, we evaluated the actual FDR of a DEG list detected in simulated small samples, according to the predefined DEGs. Based on the simulated results, using SAM with 0.05% FDR control, we tentatively defined the DEGs obtained from the full samples as a nominal gold standard set [28]. The procedure outputs totaled 3277 DEGs. Although, there were false positives in the selected DEGs, this was just a preliminary procedure which was prepared for the subsequent functional analysis of various data source integration.

### Generation of ESC Differentiation Associated Genes

**Figure 1:**
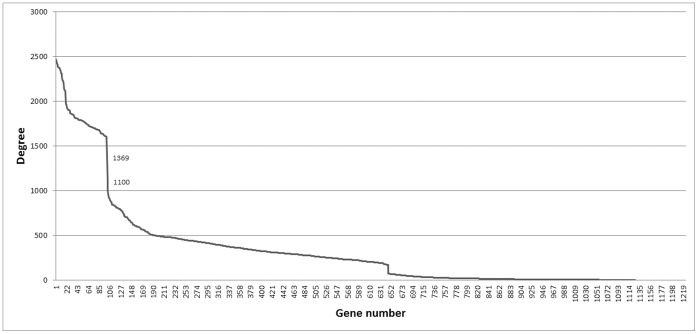
The relationship between degree and gene number. A line chart can show the relationship between the degree and gene number. Abscissa represents the gene number, and ordinate represents the degree. Bmi1 has the lowest degree, which is at the corner of the line chart. With the increase in gene number, there is a decrease in degree.

Different kinds of datasets can supply us different information, which can improve the prediction performance. In our method, each of the four score matrixes had been scaled between 0 and 1, and their combination was a merging process based on the previous DEGs selection result. That is, each dataset contained 3277 dimensions. The score between two genes which had no relation was denoted by 0. Next, we selected the final network based on the combination result. The final network was built with the genes whose scores were larger than the 75% quantile of the total score values (Formula 7). A line chart showed the relationship between the degree and the gene numbers (Figure 1). An increase in gene number resulted in a significant decrease in degree. A significant drop in degree in the graph threshold was selected for analysis. The 100th gene, Bmi1 had a degree of 1369, while the 101th gene, Tmcc3had a degree of 1100. Tmcc3 is not associated with differentiation; hence we selected the top 100 genes with the highest degrees as differentiation associated candidate genes (Table S1). The cutoff for selecting differentiation genes in the integrated network is set as 75%. 75% was the highest cutoff that included all Nanog, Poutf1 and Sox2 in the selected group. If the cutoff value was raised from this, however, Pou5f1 and Sox2 were excluded from the selected group.

Among the 100 candidate genes, 30 genes had been reported to be associated with stem cell differentiation processes (Table 1). Briefly, 17 genes tended to be significantly active in the pluripotent state and became inactive or repressed during differentiation. 13 genes tended to be significantly inactive in pluripotent state and became active or expressed during differentiation. The other 70 genes were listed in Table S2.

**Table 1:** 30 differentiation associated genes selected by weighted graph strategy.

Gene	Degree	Roles	Expression	Tissues/cells	PMID/References
Aire	1889	*+	↓	endoderm	20226168 [29]
App	1776	+	↓	neuron	18535156 [30];17908039 [31]
Bmi1	1369	+	↓	mammary stem cells	18635350 [32]
Brca1	1689	+	↓;↑	ESCs; mammary stem cells	19340312 [33];18230721 [34]
Carm1	1786	*+	↓	ESCs	19544422 [35]
Cd24a	1606	+	↑	hepatic progenitor cells; ESCs->brain,liver	17641245 [36];19998061 [37]
Cdh1	2233	+	↑	ESCs; neural stem cells->neuron	20473026 [38];19918205 [39]
Cdx2	1782	*+	↑	trophectoderm	16325584 [40]
Cyr61	1698	+	↑	neuronal differentiation; endoderm/mesoderm differentiation	9832196 [41]; 19544440 [42]
Eed	2310	*+	↓	ESCs	11803473 [43];21540835 [44]
Ids	2382	+	↑	epithelial cells	9737997 [45]
Ilk	2468	*+	↑	ESCs->cardiomyogenic differentiation	21344393 [46];22666394 [47]
Irs1	2407	*+	↓	ESCs	17620314 [48]
Irx3	1898	+	↑	ESCs->neuronal cells	21710438 [49];15611653 [50]
Klf4	2312	+	↓	monocyte differentiation	17762869 [51]
Lrp4	2275	+	↑	cardiovascular formation	15699019 [52]
Nanog	2443	*+	↓	ESCs->embryonic ectoderm	19544440 [42];22482508 [53]
Nr0b1	1642	*+	↓	individual germ layer fates	16466956 [54]
Npdc1	2241	+	↑	neural and glial precursors	9181131 [55]
Pin4	2110	+	↑	plant embryogenesis	19000164 [56]
Pou5f1	2353	*+	↑;↓	ESCs->mesoderm, ectoderm; neuronal differentiation	10742100 [57];15615706 [58]
Prc1	2122	+	↓	three germ layers	20123906 [59]
Prnp	2389	+	↓	Neuronal differentiation	10617928 [60]
Psen1	2375	+; *+	↓	ESCs->endothelial cell lineage; neuronal lineage	16376112 [61];20484632 [62]
Ptk7	1898	+	↓	expressed in un-differentiated ESC	17671748 [63]
Rap1gds1	2237	*+	↑	colony formation	20039365 [64]
Satb1	1792	*+	↑	early erythroid differentiation	15618465 [65];19933152 [66]
Sfrp2	2343	*+	↓	mesenchymal stem cells; ESCs-> dopamine neuron;ESCs->mesoderm	20826809 [67];22290867 [68];17462603 [69]
Sox2	2421	*+	↑↓	neuronal differentiation;ESCs->mesoderm	21663792 [70]
Stat3	2375	+	↓	mesoderm and endoderm differentiation	19544440 [42]

Gene: gene symbols; Degree: the degree of aim gene in the final network;Roles: the role of aim gene in the stem cell,

* represents the aim gene plays a role in maintaining stem cell pluripotency,

+represents the aim gene plays a role in stem cell differentiation process; Expression: the trend of expression level of aim gene,

↓represents a decreasing expression in differentiation,

↑represents an increasing expression in differentiation; Tissues/cells: the tissue or cells where the differentiation occurs; PMID/References: the pubmed ID of supporting published works (www.pubmed.org) and the references means the citation number in this work.

### Comparisons with Three Other Different Methods

**Figure 2:**
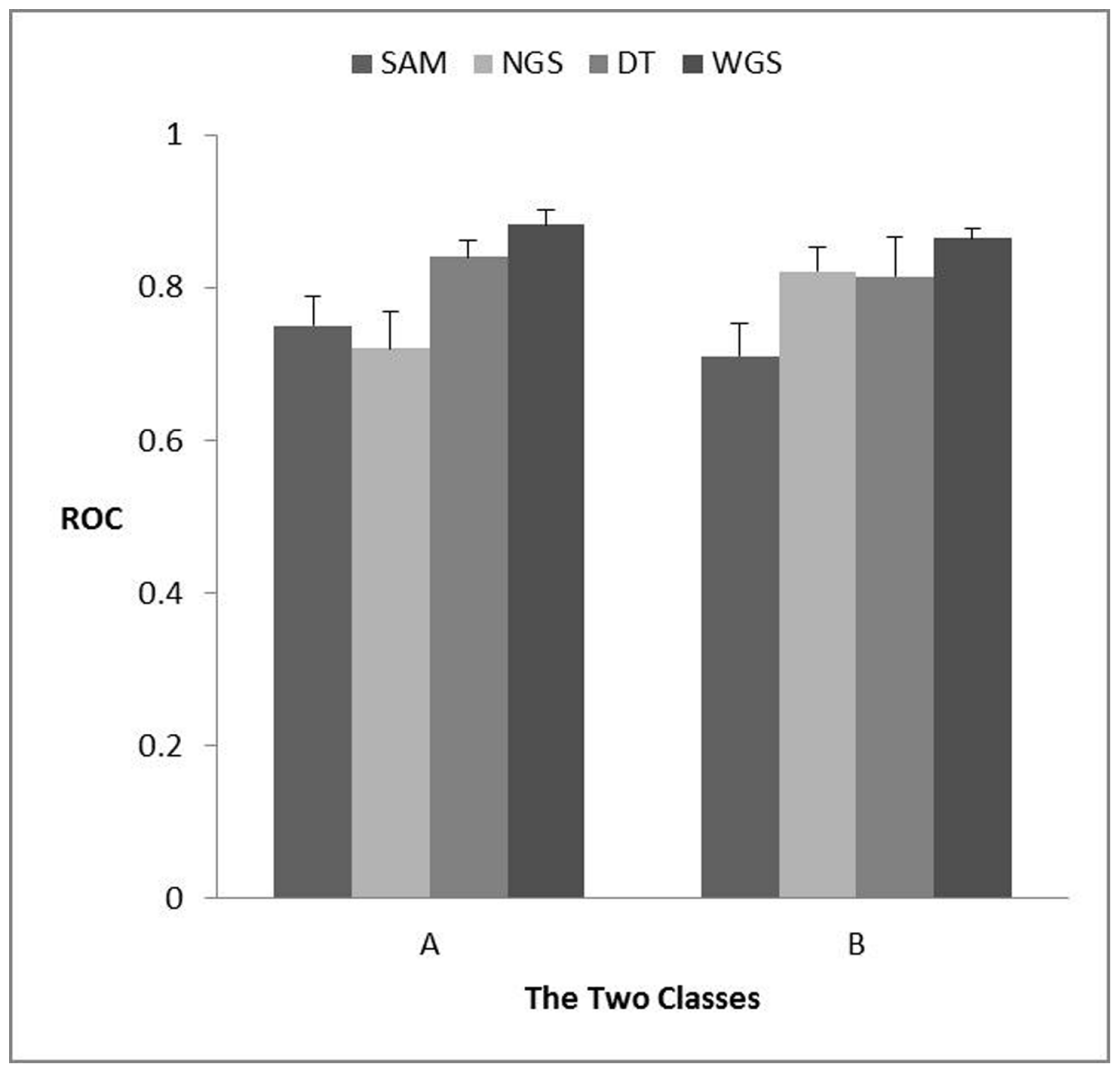
Average ROC scores for predicting the 2 classes. (Class A: the selected 30 genes; Class B: the other genes) using the 4 different approaches: (1) SAM; (2) Normal graph strategy (NGS); (3) Decision Tree (DT); (4) weighted graph strategy (WGS).

The SAM (only using the mRNA expression data set), normal graph strategy (NGS), decision tree (DT) and weighted graph strategy (WGS) were compared using the 30 differentiation associated genes as a positive gene set. Figure 2 showed the averaged Receiver Operating Characteristics (ROC) for the 30 differentiation associated genes predicted using SAM, NGS, DT and WGS. WGS took less time to make a better performance than the other three methods, and was especially easy to be understood and accepted.

### The Evaluation of Our Weighted Graph Strategy (WGS)

Differentiation associated genes were selected based on their high connectivities. The selection rule in WGS was based on the degree rather than the integrated score. This could avoid the score bias of specific datasets. There were less than 30 differentiation associated genes if the selection was based on integrated score.

## Discussion

WGS supplied a simple but reliable method to search for differentiation associated genes. Although some genes had different expression styles in different cells, the 30 genes we listed were associated with differentiation occurring not only in ESCs but also other stem cells, such as hepatic progenitor cells, plant stem cells, and neural stem cells.

We found the GO function similarity scores were higher than the sequence similarity scores, but lower than the expression scores. That was because different types of data source reflect different nature of functional relevance. As a whole, the scores of mRNA expression were always higher than others. However, the expression data might not have a higher reliability than other data sources. In order to get four balanced score matrixes, a simple weighted strategy was applied here. Firstly, the scores must be scaled between 0 and 1. Secondly, a coefficient was added into the formula. Because the scores of the other three datasets were generally lower than the expression similarity scores, a different coefficient was added in different scores, which was based on the character of dataset. For example, an average of 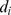 and 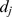 was assigned as the weight of 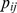. The weighted coefficient for sequence similarities was assigned as 1. Our results showed that this treatment could balance the scores, and reduced the data bias.

Weighted graph strategy based on our analysis is more efficient than SAM, DT and NGS. Firstly, weighted strategy could avoid the experimental technical biases of the derivation of different datasets according to the data character (Figure 2). Secondly, the integrated scores were used for constructing the integrated network, and the differentiation associated genes were selected based on the rank of degree in the final network.

Our weighted graph strategy was a simple but reliable method to search for differentiation associated genes. Moreover, it provided a novel way to discover candidate features associated with cell fates. Our strategy was intuitive and could be easily scaled up to for both diverse and large quantities of rapidly growing information. It could also utilize the cross genome information to further improve prediction performance. In addition, the candidate features identified in our work will be helpful in understanding the physiological processes of stem cell differentiation.

## Supporting Information

Table S1 **List of the top 100 genes selected by weighted graph strategy.**
(DOCX)

Table S2 **Functions of 70 “differentiation candidate genes” in stem cells.**
(DOCX)
