# Supplementary material for: An Efficient Weighted Graph Strategy to Identify Differentiation Associated Genes in Embryonic Stem Cells
Source: PLoS One. 2013 Apr 26;8(4):e62716. doi: 10.1371/journal.pone.0062716 (PMC3637163; doi:10.1371/journal.pone.0062716)
Supplement: Table S1 — List of the top 100 genes selected by weighted graph strategy. (DOCX) [file pone.0062716.s001.docx]

**Supplementary I**

Table S1. List of the top 100 genes selected by weighted graph strategy

| Gene | Degree | Gene | Degree | Gene | Degree | Gene | Degree |
| --- | --- | --- | --- | --- | --- | --- | --- |
| Aire | 1889 | Limd1 | 2293 | Pet112l | 2365 | Rap1gds1 | 2237 |
| App | 1776 | Lpgat1 | 2398 | Pias3 | 2363 | Rfxap | 2287 |
| Bmi1 | 1369 | Lrp4 | 2275 | Pin4 | 2110 | Rmnd1 | 2224 |
| Brca1 | 1689 | Lrrc2 | 2803 | Pls3 | 2429 | Rnf122 | 2404 |
| Carm1 | 1786 | Nanog | 2443 | Pnkd | 2275 | Rnf181 | 2270 |
| Cd24a | 1606 | Naprt1 | 2389 | Pnkp | 2471 | Rnf34 | 2416 |
| Cdh1 | 2233 | Ncapd3 | 2313 | Pnpo | 2302 | Rnf44 | 2229 |
| Cdx2 | 1782 | Nfkb1 | 2336 | Pola1 | 2454 | Rnpepl1 | 2414 |
| Cyr61 | 1698 | Nfu1 | 2381 | Polr2e | 2386 | Rpa1 | 2403 |
| Eed | 2310 | Nfyb | 2408 | Pou5f1 | 2353 | Rpn2 | 2209 |
| Hibadh | 2338 | Nol9 | 2209 | Ppcdc | 2232 | Rprm | 2464 |
| Hivep3 | 2241 | Nope | 2412 | Ppm2c | 2313 | Rpusd3 | 2308 |
| Htatip2 | 2452 | Npdc1 | 2241 | Prc1 | 2122 | Ryr1 | 2235 |
| Idh3g | 2325 | Nr0b1 | 1642 | Prepl | 2286 | Saal1 | 2302 |
| Ids | 2382 | Nudt18 | 2565 | Prkcbp1 | 2563 | Samm50 | 2406 |
| Ilk | 2468 | Osbpl1a | 2446 | Prnp | 2389 | Satb1 | 1792 |
| Irs1 | 2407 | Osbpl7 | 2276 | Prpf38a | 2219 | Sdad1 | 2374 |
| Irx3 | 1898 | Osgep | 2392 | Prpsap2 | 2428 | Selk | 2319 |
| Klf4 | 2312 | Otud7b | 2513 | Prune | 2321 | Sfrp2 | 2343 |
| L2hgdh | 2208 | P2ry5 | 2460 | Psen1 | 2375 | Sfrs17b | 2348 |
| L3mbtl2 | 2446 | Papd1 | 2351 | Psmc6 | 2262 | Sft2d2 | 2286 |
| Lactb2 | 2367 | Pdcl2 | 2731 | Ptk7 | 1898 | Sidt2 | 2518 |
| Iqcg | 2564 | Pdhx | 2384 | Ptpla | 2294 | Sox2 | 2421 |
| Ldoc1l | 2345 | Pdxk | 2342 | R3hcc1 | 2275 | Stat3 | 2375 |
| Lima1 | 2501 | Perp | 2295 | Rab5b | 2310 | Tpp1 | 2317 |
