# Supplementary material for: An Efficient Weighted Graph Strategy to Identify Differentiation Associated Genes in Embryonic Stem Cells
Source: PLoS One. 2013 Apr 26;8(4):e62716. doi: 10.1371/journal.pone.0062716 (PMC3637163; doi:10.1371/journal.pone.0062716)
Supplement: Table S2 — Functions of 70 “differentiation candidate genes” in stem cells. (DOCX) [file pone.0062716.s002.docx]

**Supplementary II**

Table S2. Functions of 70 “differentiation candidate genes” in stem cells

| Gene | Degree | Name | Chr | Location |
| --- | --- | --- | --- | --- |
| Hibadh | 2338 | 3-hydroxyisobutyrate dehydrogenase | 6 | 52546228-52640389 |
| Hivep3 | 2241 | human immunodeficiency virus type I enhancer binding protein 3 | 4 | 119733784-120138045 |
| Htatip2 | 2452 | HIV-1 tat interactive protein 2, homolog (human) | 7 | 49759106-49773999 |
| Idh3g  L2hgdh | 2325  2208 | isocitrate dehydrogenase 3 (NAD+), gamma | X | 73778963-73786897 |
|  |  | L-2-hydroxyglutarate dehydrogenase | 12 | 69690436-69724874 |
| L3mbtl2 | 2446 | l(3)mbt-like 2 (Drosophila) | 15 | 81663889-81688315 |
| Lactb2 | 2367 | lactamase, beta 2 | 1 | 13624845-13660546 |
| Iqcg | 2564 | IQ motif containing G | 16 | 33015385-33056218 |
| Ldoc1l | 2345 | leucine zipper, down-regulated in cancer 1-like | 15 | 84553398-84557823 |
| Lima1 | 2501 | LIM domain and actin binding 1 | 15 | 99778470-99875428 |
| Limd1 | 2293 | LIM domains containing 1 | 9 | 123478701-123521548 |
| Lpgat1 | 2398 | lysophosphatidylglycerol acyltransferase 1 | 1 | 191717834-191784255 |
| Lrrc2 | 2803 | leucine rich repeat containing 2 | 9 | 110951545-110984066 |
| Naprt1 | 2389 | nicotinate phosphoribosyltransferase domain containing 1 | 15 | 75890964-75894481 |
| Ncapd3 | 2313 | non-SMC condensin II complex, subunit D3 | 9 | 27030175-27095311 |
| Nfkb1 | 2336 | nuclear factor of kappa light polypeptide gene enhancer in B cells 1, p105 | 3 | 135584655-135691547 |
| Nfu1 | 2381 | NFU1 iron-sulfur cluster scaffold homolog (S. cerevisiae) | 6 | 87009236-87028461 |
| Nfyb | 2408 | nuclear transcription factor-Y beta | 10 | 82748701-82764144 |
| Nol9 | 2209 | nucleolar protein 9 | 4 | 152039321-152061494 |
| Nope | 2412 | immunoglobulin superfamily, DCC subclass, member 4 | 9 | 65101495-65137943 |
| Nudt18 | 2565 | nudix (nucleoside diphosphate linked moiety X)-type motif 18 | 14 | 70577847-70582571 |
| Osbpl1a | 2446 | oxysterol binding protein-like 1A | 18 | 12755314-12941841 |
| Osbpl7 | 2276 | oxysterol binding protein-like 7 | 11 | 97050628-97068904 |
| Osgep | 2392 | O-sialoglycoprotein endopeptidase | 14 | 50906478-50924893 |
| Otud7b | 2513 | OTU domain containing 7B | 3 | 96104527-96161129 |
| P2ry5 | 2460 | lysophosphatidic acid receptor 6 | 14 | 73237895-73243294 |
| Papd1 | 2351 | mitochondrial poly(A) polymerase | 18 | 4375592-4397330 |
| Pdcl2 | 2731 | phosducin-like 2 | 5 | 76312115-76331156 |
| Pdhx | 2384 | pyruvate dehydrogenase complex, component X | 2 | 103021075-103073513 |
| Pdxk | 2342 | pyridoxal (pyridoxine, vitamin B6) kinase | 10 | 78436744-78464975 |
| Perp | 2295 | PERP, TP53 apoptosis effector | 10 | 18845071-18857073 |
| Pet112l | 2365 | PET112 homolog (S. cerevisiae) | 3 | 85574119-85655622 |
| Pias3 | 2363 | protein inhibitor of activated STAT 3 | 3 | 96696384-96706070 |
| Pls3 | 2429 | plastin 3 (T-isoform) | X | 75785654-75875182 |
| Pnkd | 2275 | paroxysmal nonkinesiogenic dyskinesia | 1 | 74284930-74353694 |
| Pnkp | 2471 | polynucleotide kinase 3'- phosphatase | 7 | 44857139-44862925 |
| Pnpo | 2302 | pyridoxine 5'-phosphate oxidase | 11 | 96937825-96943986 |
| Pola1 | 2454 | polymerase (DNA directed), alpha 1 | X | 93304767-93632155 |
| Polr2e | 2386 | polymerase (RNA) II (DNA directed) polypeptide E | 10 | 80035949-80039795 |
| Ppcdc | 2232 | phosphopantothenoylcysteine decarboxylase | 9 | 57412668-57440114 |
| Ppm2c | 2313 | pyruvate dehyrogenase phosphatase catalytic subunit 1 | 4 | 11958184-11966452 |
| Prepl | 2286 | prolyl endopeptidase-like | 17 | 85063477-85090267 |
| Prkcbp1 | 2563 | zinc finger, MYND-type containing 8 | 2 | 165784155-165899016 |
| Prpf38a | 2219 | PRP38 pre-mRNA processing factor 38 (yeast) domain containing A | 4 | 108563172-108579336 |
| Prpsap2 | 2428 | phosphoribosyl pyrophosphate synthetase-associated protein 2 | 11 | 61729654-61762062 |
| Prune | 2321 | prune homolog (Drosophila) | 3 | 95253674-95282076 |
| Psmc6 | 2262 | proteasome (prosome, macropain) 26S subunit, ATPase, 6 | 14 | 45329824-45349071 |
| Ptpla | 2294 | protein tyrosine phosphatase-like (proline instead of catalytic arginine), member a | 2 | 13975563-14056023 |
| R3hcc1 | 2275 | R3H domain and coiled-coil containing 1 | 14 | 69697307-69707584 |
| Rab5b | 2310 | RAB5B, member RAS oncogene family | 10 | 128677193-128696268 |
| Rfxap | 2287 | regulatory factor X-associated protein | 3 | 54803116-54807791 |
| Rmnd1 | 2224 | required for meiotic nuclear division 1 homolog (S. cerevisiae) | 10 | 4401915-4432388 |
| Rnf122 | 2404 | ring finger protein 122 | 8 | 31111820-31131482 |
| Rnf181 | 2270 | ring finger protein 181 | 6 | 72359714-72362721 |
| Rnf34 | 2416 | ring finger protein 34 | 5 | 122850188-122868945 |
| Rnf44 | 2229 | ring finger protein 44 | 13 | 54679401-54693907 |
| Rnpepl1 | 2414 | arginyl aminopeptidase (aminopeptidase B)-like 1 | 1 | 92910783-92924384 |
| Rpa1 | 2403 | replication protein A1 | 11 | 75298166-75348324 |
| Rpn2 | 2209 | ribophorin II | 2 | 157279017-157326319 |
| Rprm | 2464 | reprimo, TP53 dependent G2 arrest mediator candidate | 2 | 54084093-54085552 |
| Rpusd3 | 2308 | RNA pseudouridylate synthase domain containing 3 | 6 | 113415319-113419348 |
| Ryr1 | 2235 | ryanodine receptor 1, skeletal muscle | 7 | 29003340-29125151 |
| Saal1 | 2302 | serum amyloid A-like 1 | 7 | 46686108-46710680 |
| Samm50 | 2406 | sorting and assembly machinery component 50 homolog (S. cerevisiae) | 15 | 84192233-84214303 |
| Sdad1 | 2374 | SDA1 domain containing 1 | 5 | 92284010-92310024 |
| Selk | 2319 | selenoprotein K | 14 | 29968308-29975074 |
| Sfrs17b | 2348 | A kinase (PRKA) anchor protein 17B | X | 36608314-36645395 |
| Sft2d2 | 2286 | SFT2 domain containing 2 | 1 | 165174341-165194433 |
| Sidt2 | 2518 | SID1 transmembrane family, member 2 | 9 | 45937857-45955247 |
| Tpp1 | 2317 | tripeptidyl peptidase I | 7 | 105744847-105752207 |

Chr: Chromosome.

Location: the position of a gene on a chromosome.
